# Simple immobilization for stereotactic radiotherapy aimed at pelvic metastases

**DOI:** 10.1016/j.phro.2023.100460

**Published:** 2023-06-20

**Authors:** Jorinde Janssen, Charlotte L. Brouwer, Floor H.E. Staal, Heleen E. van Herpt, Stefan Both, Johannes A. Langendijk, Shafak Aluwini

**Affiliations:** Department of Radiation Oncology, University of Groningen, University Medical Center Groningen, Groningen, the Netherlands

**Keywords:** Immobilization, SBRT, Pelvis, Oligometastasis, PCa

## Abstract

Stereotactic body radiotherapy (SBRT) is increasingly applied for pelvic oligometastases of prostate cancer, and currently no simple immobilization method is available for cone beam computed tomography (CBCT)-guided treatment. We assessed patient set-up and intrafraction motion using simple immobilization during CBCT-guided pelvic SBRT. Forty patients were immobilized with basic arm- head- and knee support and either a thermoplastic cushion or a foam cushion. Analysis of 454 CBCTs showed mean intrafraction translation <3.0 mm in 94% of fractions and mean intrafraction rotation <1.5° in 95% of fractions. Therefore, simple immobilization ensured stable patient positioning during CBCT-guided pelvic SBRT.

## Highlights

• Simple immobilization resulted in acceptable patient stability.
• Patient intrafraction translation and treatment time were not correlated in this cohort.
• Two different positioning cushions showed similar patient stability.

## 1 Introduction

Image guided stereotactic body radiotherapy (SBRT) is an important treatment option for prostate cancer (PCa) patients [[1], [2], [3], [4]]. SBRT targeting a limited number of PCa metastases (oligometastases) has shown promising results concerning progression-free and overall survival [[3], [4], [5]]. PCa oligometastases mostly concern lymph node or skeletal lesions located in the pelvis [[6], [7]]. Consequently, metastasis-directed radiotherapy aimed at pelvic metastases has been increasingly used [8]. SBRT is used to precisely deliver high radiation dose to the tumour in few fractions, using tight margins and steep dose gradients to minimize side effects to surrounding organs at risk. Therefore, delivering high quality SBRT requires reliable patient positioning and immobilization tools. Influence of immobilization on patient set-up and intrafraction motion has been widely reported for targets such as intra-cranial, spinal, lung and liver using sophisticated and time consuming immobilization methods, including vacuum mattresses, thermoplastic masks, and body lock frame systems [[9], [10], [11], [12]]. However, a simple immobilization method for SBRT to pelvic lesions has not been described thus far.

Werensteijn et al. reported that vacuum fixation could be omitted for MRI-guided SBRT to lymph node lesions [13]. However, data reporting the use of simple immobilization devices for cone beam computed tomography (CBCT)-guided SBRT are scarcely available and report on a limited number of patients involving only part of the whole treatment [14].

Therefore, this study, concerning SBRT for pelvic oligometastatic lesions, aimed to assess the effect of simple immobilization on patient set-up and intrafraction motion, assess the correlation between intrafraction motion and treatment time per fraction, and compare the use of two different positioning cushions regarding their effect on set-up and intrafraction motion.

## 2 Materials and methods

CBCT data of 40 consecutive PCa patients treated between April 2020 and December 2021 with SBRT to pelvic oligo-recurrent lesions were used to analyse patient positioning and intrafraction motion. This data included a total of 454 CBCTs (196 fractions) consisting of 196 pre-fraction CBCTs, 61 repeat pre-fraction CBCTs, 16 intrafraction CBCTs, and 180 post-fraction CBCTs. All patients were included in the ADOPT clinical trial (NCT04302454 [15]) and provided written informed consent for the use of their data. Lymph node lesions (38 patients, 53 lesions) were mostly located near the iliac vessels (77%) and the obturator area (15%). Both bone lesions (2 patients) were located in the iliac bone.

### 2.1 Immobilization

Patients were immobilized with arm- and head support (Posirest™-2, Cablon Medical, Leusden NL), knee support (Kneefix™ 3 with elevation block and lockbar, CIVCO Radiotherapy, Iowa USA) and feet fixation (Feetfix™ 3 with lockbar, CIVCO Radiotherapy, Iowa USA). No standard immobilization cushion was available for this indication and two cushions available within our centre were selected for this clinical pilot analysis. Ten patients were immobilized with a thermoplastic cushion (Klarity Cushion™ R550-L2, Klarity Medical Products, Ohio USA) with the intend to be compared to a foam cushion (Couchtop™ Cover, CIVCO Radiotherapy, Iowa USA), which was used for immobilization of the following 30 patients. The thermoplastic cushion (TC) was a customized support cushion of 60 cm in length that was moulded to the individual patient shape. The foam cushion (FC) was a re-usable thin mattress (Supplementary Material S1). Patient age, length, and weight were balanced between the two cushion groups (Supplementary Material S2).

### 2.2 Set-up

Patient set-up to the isocentre was done using patient markings (tattoo points) and lasers. Online acquired 3D-CBCT images were registered to the planning CT using automatic image registration (Elekta XVI, Elekta Solutions Ab, Stockholm SWE). The registration mask included the pelvic bone structures adjacent to the planning target volume.

Patients were repositioned in case of rotation >5.0 degrees, followed by a new pre-fraction CBCT. Couch translations were executed by our 3D couch (Elekta NPSS, Elekta Solutions Ab, Stockholm SWE) in left-right (LR), anterior-posterior (AP) and superior-inferior (SI) direction. If correction exceeded 3.0 mm in any direction, a repeat pre-fraction CBCT was performed to ensure correct patient position after correction.

### 2.3 Treatment planning and delivery

Patients were treated with either 3 fractions of 10 Gy (bone lesions) or 5 fractions of 7 Gy (lymph node lesions) conform the ADOPT trial protocol [15]. The applied CTV – PTV margin was 5.0 mm.

Treatment plans consisted of 2 VMAT arcs (Elekta Synergy®, Elekta Solutions Ab, Stockholm SWE). An in between-arc CBCT (intrafraction CBCT) was applied during the first fraction in case of long delivery time or lesion location close to moving organs at risk. Translational deviations >1.0 mm intrafraction CBCT obligated a translational correction before the start of the second arc, and these fractions were excluded from the intrafraction motion analysis to avoid bias introduced by repositioning (4 fractions). After completion of arc delivery, the final CBCT was performed (post-fraction CBCT). In 16/196 fractions (8%), post-fraction CBCT was missing or incorrectly registered, and these fractions were excluded from the intrafraction motion analysis. At least 3 post-fraction CBCTs were available for all patients.

### 2.4 Analysis

Image registration data were extracted from TheraView (Cablon Medical, Leusden NL), data analysis was performed using SPSS® Statistics 28 (IBM®, New York USA). Registration results were analysed for pre-, repeat pre-, intra-, and post-fraction CBCTs, reporting the means and standard deviations (SD). The systematic error (Σ) was calculated as the SD of the mean translation per treatment plan and the random error (σ) as the root-mean square of the SD per treatment plan [16]. Statistical tests used concerned the unpaired *T*-test (normally distributed demographics), Fisher’s exact test (frequencies), and the Mann-Whitney *U* test (absolute translations and rotations). Additionally, the time between the start of the pre-fraction CBCT and the start of the post-fraction CBCT (minutes) was extracted and related to the associated translations to assess correlation (Spearman’s rank-order correlation).

## 3 Results

### 3.1 Initial positioning

The mean (±SD) patient set-up translations were 0.1 (±2.4) mm LR, −0.4 (±2.6) mm SI, and 0.1 (±2.3) mm AP. During pre-fraction CBCT, the set-up translations were ≤3.0 mm in 83% of fractions LR, 79% of fractions SI, and 89% of fractions AP. Rotational set-up displacements were less than ≤3.0 degrees in 95% of fractions for pitch, 100% of fractions for yaw, and 100% of fractions for roll (Table 1). Rotational set-up displacement >5.0 degrees occurred in only 1/196 fractions (0.5%) and concerned the pitch direction.

**Table 1:** A: Mean (±SD) and absolute mean (±SD) patient set-up translations and rotations. B: Mean (±SD), absolute mean (±SD), systematic error and random errors of patient intrafraction translations and rotations.

A) Patient set-up					
		Total	Thermoplastic Cushion	Foam Cushion	P-value*
**Translations (mm)**					
**LR**	Mean (±SD)	0.1 (±2.4)	0.2 (±2.0)	0.1 (±2.5)	
	Absolute mean (±SD)	1.8 (±1.5)	1.5 (±1.2)	1.9 (±1.6)	0.19
**SI**	Mean (±SD)	−0.4 (±2.6)	−0.7 (±2.5)	−0.3 (±2.7)	
	Absolute mean (±SD)	2.0 (±1.8)	2.1 (±1.6)	2.0 (±1.8)	0.35
**AP**	Mean (±SD)	0.1 (±2.3)	0.4 (±2.8)	0.0 (±2.1)	
	Absolute mean (±SD)	1.6 (±1.6)	**2.2 (±1.8)**	**1.4 (±1.5)**	**0.00**

**Rotations (degrees)**					
**Pitch**	Mean (±SD)	−0.2 (±1.4)	−0.2 (±1.5)	−0.2 (±1.4)	
	Absolute mean (±SD)	1.0 (±1.0)	1.1 (±1.0)	1.0 (±1.0)	0.49
**Yaw**	Mean (±SD)	−0.2 (±0.9)	−0.2 (±0.6)	−0.2 (±0.9)	
	Absolute mean (±SD)	0.7 (±0.6)	**0.5 (±0.4)**	**0.7 (±0.6)**	**0.05**
**Roll**	Mean (±SD)	−0.1 (±0.6)	−0.2 (±0.5)	−0.1 (±0.6)	
	Absolute mean (±SD)	0.4 (±0.4)	0.4 (±0.4)	0.4 (±0.4)	0.76

B) Intrafraction motion					
		Total	Thermoplastic Cushion	Foam Cushion	P-value*
**Translations (mm)**					
**LR**	Mean (±SD)	−0.1 (±1.0)	−0.0 (±0.8)	−0.1 (±1.1)	
	Absolute mean (±SD)	0.7 (±0.8)	**0.5 (±0.6)**	**0.7 (±0.9)**	**0.02**
	Systematic error	0.6	0.2	0.7	
	Random error	1.0	0.8	1.0	
**SI**	Mean (±SD)	−0.1 (±0.6)	−0.2 (±0.6)	−0.1 (±0.7)	
	Absolute mean (±SD)	0.5 (±0.5)	0.4 (±0.5)	0.5 (±0.5)	0.12
	Systematic error	0.40	0.31	0.43	
	Random error	0.57	0.54	0.58	
**AP**	Mean (±SD)	0.3 (±0.7)	0.3 (±0.4)	0.3 (±0.7)	
	Absolute mean (±SD)	0.5 (±0.5)	0.4 (±0.4)	0.5 (±0.5)	0.29
	Systematic error	0.44	0.32	0.48	
	Random error	0.59	0.41	0.64	

**Rotations (degrees)**					
**Pitch**	Mean (±SD)	0.0 (±0.6)	−0.1 (±0.7)	0.1 (±0.5)	
	Absolute mean (±SD)	0.4 (±0.4)	0.4 (±0.5)	0.4 (±0.4)	0.72
	Systematic error	0.42	0.52	0.37	
	Random error	0.49	0.63	0.45	
**Yaw**	Mean (±SD)	0.0 (±0.6)	0.2 (±0.6)	0.1 (±0.6)	
	Absolute mean (±SD)	0.4 (±0.4)	0.4 (±0.4)	0.4 (±0.4)	0.43
	Systematic error	0.31	0.32	0.31	
	Random error	0.53	0.51	0.54	
**Roll**	Mean (±SD)	0.0 (±0.3)	0.0 (±0.4)	0.0 (±0.3)	
	Absolute mean (±SD)	0.3 (±0.2)	0.3 (±0.3)	0.2 (±0.2)	0.65
	Systematic error	0.24	0.28	0.23	
	Random error	0.28	0.30	0.28	

*Mann-Whitney U Test (two sided, exact) for absolute values (bold in case of p < 0.05).

*Left-right (LR), Superior-inferior (SI), Anterior-posterior (AP)*.

Repeat pre-fraction CBCT (performed if correction >3 mm was necessary) showed a translational displacement <1.0 mm in all directions in 80% (49 / 61 fractions).

Patients immobilized with TC showed significantly larger absolute translations in AP compared to those immobilized with FC (2.2 mm vs. 1.4 mm, p < 0.01). On the contrary, the absolute mean rotation was smaller in the yaw direction for patients immobilized with TC compared to FC with an absolute mean of 0.5° vs. 0.7° (p = 0.05) (Table 1).

### 3.2 Intrafraction motion

The mean (±SD) translations per fraction were −0.1 (±1.0) mm LR, −0.1 (±0.6) mm SI, and −0.3 (±0.3) mm AP. The intrafraction motion (all directions) was less than 2.0 mm in 87% of fractions, and was less than 3.0 mm in 94% of fractions. Absolute mean intrafraction translation was significantly larger in LR for the FC group compared to the TC group (0.7 mm (FC) vs. 0.5 mm (TC), p = 0.02). This also resulted in a larger systematic error for immobilization with FC in this direction (0.7 vs. 0.2 mm).

Rotational intrafraction motion was <1.5° for all directions in 171 fractions (95%). Mean rotational intrafraction motion was 0.0° for all three directions with a SD <0.6° (Table 1 and Supplementary Material S3).

### 3.3 Treatment time

**Fig. 1:**
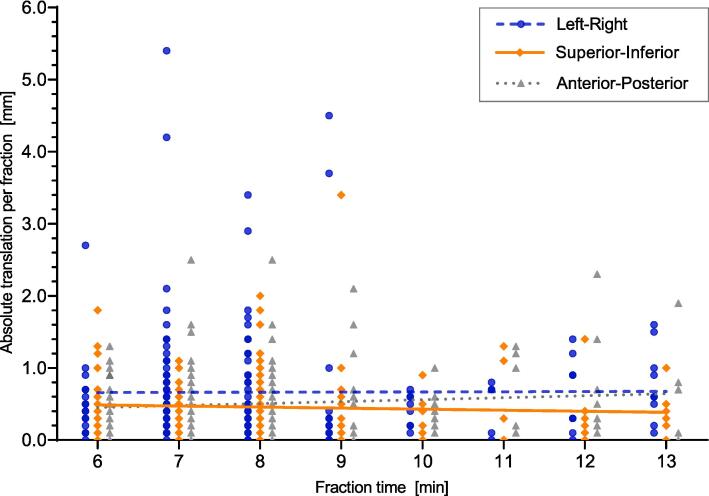
Scatterplot representing absolute translation per fraction over treatment time of treatments within 6 to 13 min (n = 173). The Spearman’s rank-order correlation was 0.01 (p = 0.89) left–right, rs = 0.13 (p = 0.10) superior-inferior, rs = −0.01 (p = 0.94) anterior-posterior.

Overall, in 180 fractions (with post-fraction CBCT present), mean (±SD) time interval between start of pre-fraction and post-fraction CBCT was 9 (±3) minutes, and the time interval was ≤13 min in 173 fractions (97%). The mean (±SD) time interval was 8 (±2) minutes for fractions where one pre-fraction CBCT was performed (n = 125) and 11 (±4) minutes in fractions with repeat pre-fraction CBCT (n = 55). Intrafraction CBCT was performed in 16 fractions, and for these 16 fractions mean time interval was increased by 6 min. Correlation analysis showed no correlation between treatment time and translational intrafraction motion, indicating no increase in patient intrafraction motion with prolonged treatment duration (Fig. 1).

## 4 Discussion

The use of simple patient immobilization in patients treated with SBRT targeting pelvic metastases resulted in mean set-up translations between −0.4 and 0.1 mm, which minimized the need to repeat pre-fraction CBCT to only 31% of fractions. Furthermore, simple immobilization resulted in a translational intrafraction motion <3.0 mm in 94% of fractions and rotational motion <1.5° in 95% of fractions. These set-up and intrafraction stability results are clinically acceptable and well within PTV margins.

To our knowledge, our results are the first with a complete analysis on the influence of simple immobilization on patient set-up and intrafraction motion during CBCT-guided SBRT to pelvic lesions. Data reporting the use of simple immobilization devices for pelvic SBRT are scarce, and currently available research is executed in MR guided SBRT or investigated only part of treatment in few patients [[13], [14]].

One of the most used immobilization methods for SBRT is vacuum fixation [[9], [10], [12], [13]]. Our mean (±SD) intrafraction translations of −0.1 (±1.0) mm LR, −0.1 (±0.6) mm SI, and −0.3 (±0.7) mm AP were comparable to MR guided lymph node SBRT using vacuum cushion, which reported mean patient intrafraction motion of 0.0 (±0.6) mm LR, −0.3 (±0.9) mm SI, and 0.2 (±0.5) mm AP during the first 15 min after positioning (comparable to our median treatment time of 13 min) [13]. Our comparable results to those reported with the use of vacuum cushion highlight the reliable stability achieved with simple immobilization for pelvic metastases. Furthermore, the simple positioning method reduced preparation time and costs compared to vacuum fixation, making the application during CBCT guided pelvic SBRT advantageous.

The importance of short treatment time in SBRT has been stressed for spinal and intra-cranial SBRT, in which a correlation between (long) treatment time and the magnitude of intrafraction translations has been reported [[17], [18]]. These correlations were found during mean treatment times of 15–70 min and might not be applicable to the relatively short treatment time needed for CBCT-guided pelvic SBRT. In the current study, no correlation between treatment time (mean 9 min) and intrafraction motion was found. Therefore, we assessed that an additional CBCT in between arc delivery would not contribute to further reduction of overall intrafraction motion and may unnecessarily prolong overall treatment time.

One of the possible drawbacks of less extensive immobilization could be an increased rate of excessive set-up rotations requiring manual repositioning and, therefore, extra overall treatment time [[19], [20]]. According to our protocol, manual repositioning after pre-fraction CBCT should be applied if >3.0° rotational displacement was registered. In this study the manual repositioning was sparsely needed since rotational displacements were ≤3.0° in 95%, 100%, and 100% (pitch, yaw, roll) of fractions.

A secondary aim of this study was to assess the effect of two different immobilization cushions on set-up and intrafraction motion. Immobilization with the TC (customized) and FC (re-usable) showed similar results in most translational and rotational directions, both during set-up and during treatment. We reported a significant difference in left–right intrafraction motion in favour of the TC with a difference of 0.2 mm in absolute mean. However, this difference is small and its clinical relevance is very limited, especially considering the margin applied in our institute (PTV margin 5.0 mm).

The next planned step for this group of patients is to investigate the effect of lesion intrafraction motion (CBCT) and isocentric precision optimization, which could enable us to reduce this PTV margin for SBRT to pelvic oligometastases.

The positioning results associated with simple positioning were of direct effect on our daily practice, reducing overall treatment time, sparing personnel time, and avoiding the use of expensive immobilization devices for this group of patients. The expanding use of stereotactic radiotherapy for oligometastatic disease underlines the preference towards simple immobilization with re-usable materials and could have a substantial impact on the environmental footprint of health care.

## Declaration of Competing Interest

The authors declare the following financial interests/personal relationships which may be considered as potential competing interests: The evaluated patient group is part of the ADOPT clinical trial (Principal Investigator S. Aluwini, NCT04302454), which has been funded by the Dutch Cancer Society (KWF, Amsterdam). The department of Radiation Oncology of the University Medical Center Groningen has research contracts with IBA, RaySearch, Siemens, Elekta, Leoni, and Mirada, and has received grants from the Dutch Cancer Society and the European Union.
