# Supplementary material for: Simple immobilization for stereotactic radiotherapy aimed at pelvic metastases
Source: Phys Imaging Radiat Oncol. 2023 Jun 20;27:100460. doi: 10.1016/j.phro.2023.100460 (PMC10331836; doi:10.1016/j.phro.2023.100460)
Supplement: Supplementary Data 1 [file mmc1.docx]

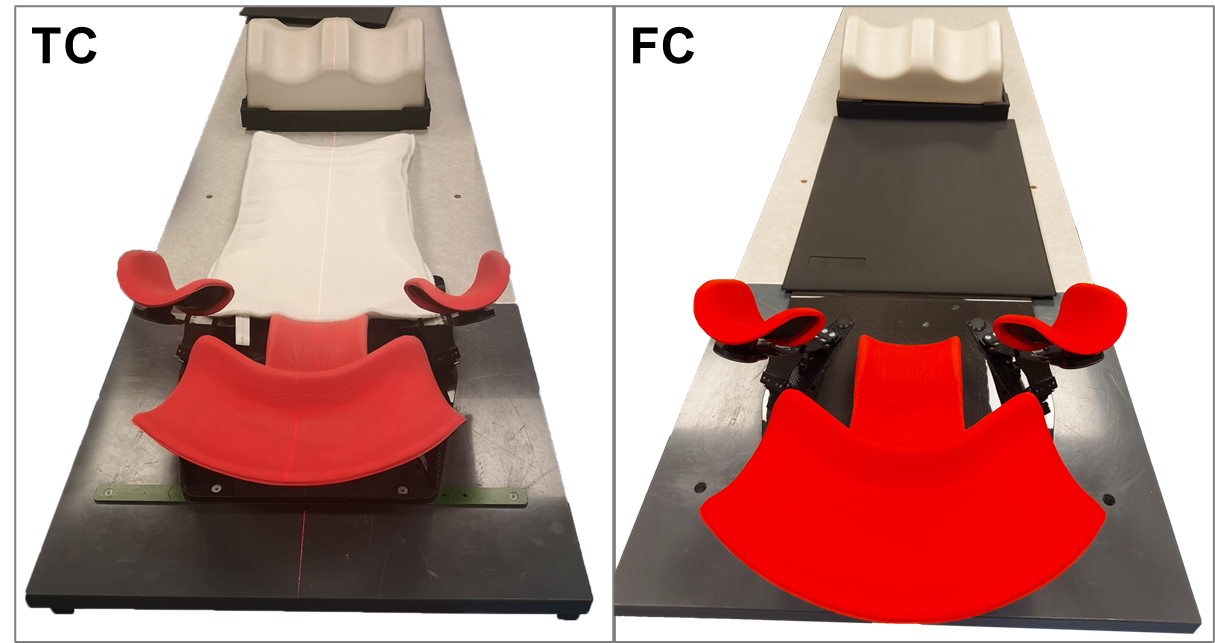


**Supplementary material S1** Immobilization cushions used in this study: the thermoplastic cushion (TC, Left) and the foam cushion (FC, Right).
