# Supplementary material for: Simple immobilization for stereotactic radiotherapy aimed at pelvic metastases
Source: Phys Imaging Radiat Oncol. 2023 Jun 20;27:100460. doi: 10.1016/j.phro.2023.100460 (PMC10331836; doi:10.1016/j.phro.2023.100460)
Supplement: Supplementary Data 2 [file mmc2.docx]

**Supplementary material S2** Demographics of 40 oligo-recurrent prostate cancer patients

| Variable |  | Total | Thermoplastic Cushion | Foam Cushion | P-value* |
| --- | --- | --- | --- | --- | --- |
| Age (years) |  | 70.3 (± 5.7) | 71.0 (± 3.9) | 70.1 (± 6.2) | 0.66 |
| Length (cm) |  | 179.3 (± 6.5) | 179.4 (± 8.1) | 179.2 (± 6.0) | 0.93 |
| Weight (kg) |  | 89.3 (± 14.0) | 92.7 (± 17.1) | 88.1 (± 12.9) | 0.41 |
| BMI (kg/m^2^) |  | 27.9 (± 4.2) | 28.6 (± 4.3) | 27.6 (± 4.2) | 0.51 |
| Fractionation | **3 x 10 Gy** | 2 (5%) | 1 (10%) | 1 (3%) |  |
|  | **5 x 7 Gy** | 38 (95%) | 9 (90%) | 29 (97%) |  |

* T-test (unpaired)
