# Supplementary material for: Simple immobilization for stereotactic radiotherapy aimed at pelvic metastases
Source: Phys Imaging Radiat Oncol. 2023 Jun 20;27:100460. doi: 10.1016/j.phro.2023.100460 (PMC10331836; doi:10.1016/j.phro.2023.100460)
Supplement: Supplementary Data 3 [file mmc3.docx]

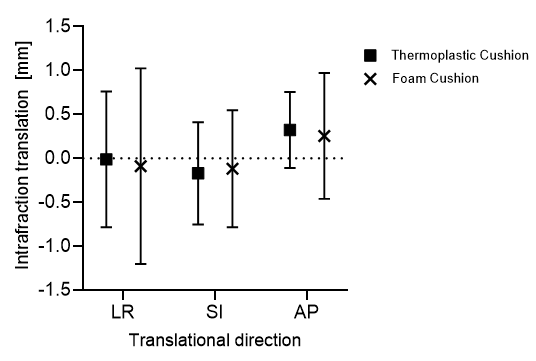


**Supplementary material S3** Mean (±SD) intrafraction translation in left-right (LR), superior-inferior (SI), and anterior-posterior (AP) for both immobilization cushions.
